# Understanding Rare Anemias: Emerging Frontiers for Diagnosis and Treatment

**DOI:** 10.3390/jcm13113180

**Published:** 2024-05-29

**Authors:** Joan-Lluis Vives Corrons

**Affiliations:** 1 Rare Anaemias and Erythropoietic Disorders Research, Institute for Leukaemia Research Josep Carreras, 08916 Barcelona, Spain; jlvives@clinic.cat; 2 Ektacytometry Unit, Clinical Centre for Ambulatory Medicine, 08036 Barcelona, Spain

**Keywords:** erythrocytes, anemia, rare diseases, diagnosis, treatment

## Abstract

**Background**—This review provides a comprehensive overview of rare anemias, emphasizing their hereditary and acquired causes, diagnostic advancements, and evolving treatment strategies. It outlines the significance of rare anemias within public health, historical challenges in recognition and treatment, and the role of European initiatives like ENERCA and EuroBloodNet in advancing care. **Content**—This document discusses diagnostic technologies like next-generation sequencing and the impact of artificial intelligence, alongside the promising avenues of gene therapy, targeted drug treatments, and stem cell transplantation. It underscores the importance of a patient-tailored approach, advances in diagnostic tools, and the necessity for continued research, patient advocacy, and international collaboration to improve outcomes for individuals with rare anemias.

## 1. Introduction

Rare anemias (RAs) represent a diverse group of rare diseases (RDs) characterized by anemia as their primary symptom. These conditions are deemed rare due to their prevalence of less than five cases per 10,000 individuals in the European demographic, with more than 80% being hereditary. Each variant exhibits unique clinical features and implications for public health [1]. In 2002, the European Commission (EC) recognized the importance of RAs through the approval of the European Network for Rare and Congenital Anemias (ENERCA), a project that facilitated the development and dissemination of the knowledge on RAs worldwide [2]. ENERCA’s initiatives for health care services and research, over two decades, have significantly enhanced the understanding and management of these conditions, culminating in the establishment of the European Reference Network (ERN) for Rare Hematological Diseases (RHD) or EuroBloodNet, which includes oncological and non-oncological diseases [3]. Interestingly, the ENERCA project started shortly before the creation of the EC High-Level Group (HLG) that in 2004 brought together experts from all the member states (MS) in several areas of RD expertise: (a) patient’s safety and quality of care, (b) health assessment and health systems, (c) health technology, (d) European workforce for health professionals, (e) information and e-health, and (f) cross-border healthcare purchasing and provision [4]. For RAs, this was a great advantage, because ENERCA facilitated the progressive development and implementation of RA knowledge and awareness and developed the required health care services in parallel to the development and progress of the different EC-HLG areas of expertise.

Prior to the establishment of ENERCA, RAs were largely unrecognized, even among healthcare professionals. This lack of recognition stemmed from the obscure causes of these anemias and the absence of effective treatments. Consequently, for many years, RAs were frequently misdiagnosed, leading to their underestimation by public health care providers. ENERCA significantly transformed this scenario through the initiation of four successive research projects spanning a total of 20 years. Today, its robust successor, the European Reference Network (ERN) for Rare Hematological Diseases (RHD), known as ERN-RHD EuroBloodNet, has adopted and implemented all resources and services developed by ENERCA from 2002 until 2017. This network has emerged as a pivotal resource for enhancing the diagnosis of RHDs and providing high-quality healthcare to all patients with RA conditions that necessitate specialized resources or expertise. Furthermore, EuroBloodNet serves as a hub for medical training and research, the dissemination of information, and the evaluation of RHDs. It plays a crucial role in establishing national contact points for RHDs, thereby contributing significantly to the field.

The classification of rare anemias into hereditary and acquired categories reflects their diverse causes, including bone marrow failure (BMF), red blood cell (RBC) destruction (hemolysis), and blood loss (bleeding). The most relevant and well-known conditions that can lead to an RA are summarized in Table 1. Recent decades have seen notable advancements in understanding and treating bone marrow failure (BMF) syndromes, with progress in delineating inherited and acquired causes. The American Society of Hematology emphasizes the diagnostic challenge BMF presents, highlighting the critical nature of distinguishing between conditions like acquired aplastic anemia and inherited BMF syndromes for optimal patient care. The treatment landscape for rare anemias is evolving, with significant progress in gene therapy, targeted drug treatments, and stem cell transplantation. Gene therapy offers a promising avenue for curing hereditary anemias like beta-thalassemia and sickle cell disease (SCD). Targeted drug treatments, such as *Mitapivat* for pyruvate kinase deficiency (PKD), represent a new era of disease-modifying agents. Hematopoietic stem cell transplantation continues to be refined, improving outcomes for conditions like Fanconi anemia (FA).

Emerging diagnostic technologies, including next-generation sequencing (NGS) and artificial intelligence, are enhancing the accuracy and efficiency of RA diagnosis. These advancements, along with improved treatment strategies, underscore a shift towards personalized medicine, promising better outcomes and quality of life for patients. The journey from the early recognition of rare anemias to the current landscape of advanced diagnostics and treatment reflects a significant evolution in the field. Continued research, patient advocacy, and international collaboration are essential to further understanding these complex conditions and developing innovative therapies. As the field progresses, it remains imperative to ensure equitable access to these advancements, addressing the challenges of rare anemias through a comprehensive and inclusive approach.

## 2. Rare Anemias Due to Bone Marrow Failure

**Figure 1:**
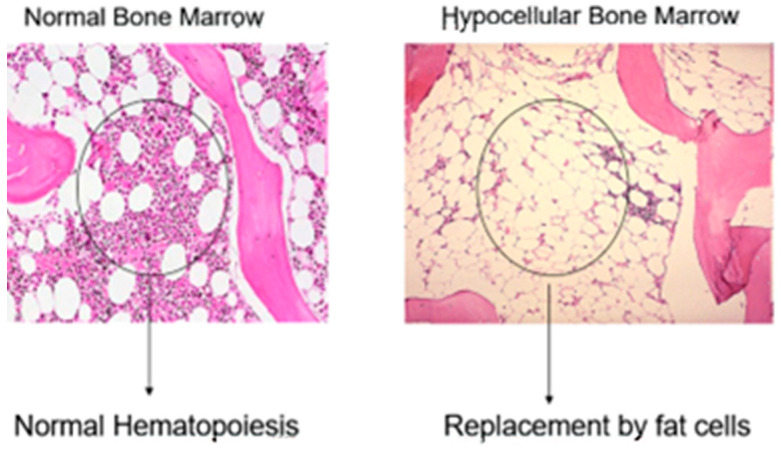
Bone marrow biopsy. Normal bone marrow cellularity (**left**) compared with severe hypoplasia (**right**).

BMF is a condition that occurs when the bone marrow fails to produce enough blood cells. It can be caused by a variety of factors, including genetic mutations, infections, and exposure to toxins. BMF can lead to a range of symptoms—including anemia, as one of its most important clinical manifestations—and signifies a substantial clinical concern. Historically, BMF syndromes have been challenging to understand and manage, often resulting in fatal outcomes. Nonetheless, research advancements over the past two decades have significantly enhanced our comprehension of these disorders, leading to therapeutic innovations with better clinical outcomes and improved patient prognoses. BMF is classified into inherited and acquired, but the distinction between acquired aplastic anemia (aAA), hypocellular myelodysplastic syndrome (MDS), and inherited BMF syndromes, all presenting with bone marrow hypocellularity (Figure 1), is critical to inform appropriate care [5]. Moreover, the clinical and laboratory evaluation of patients with suspected BMF is a critical diagnostic challenge. The overlapping clinical presentations and bone marrow characteristics of both inherited and acquired hypocellular marrow failure underscore the complexity of diagnosing these conditions.

BMF syndromes are categorized into inherited and acquired types. Accurately distinguishing among aAA, hypocellular MDS, and inherited BMF syndromes is crucial for establishing appropriate treatment protocols. According to the American Society of Hematology (ASH) Educational Program, the clinical and laboratory evaluation of patients with suspected hypocellular marrow failure poses a significant diagnostic challenge in real case scenarios, particularly in non-severe disease [5]. These challenges necessitate a comprehensive clinical and laboratory assessment to inform a nuanced approach to patient management. The ongoing refinement in understanding and treating BMF syndromes represents a significant stride towards better patient outcomes, underscoring the importance of continued research and specialized care in this domain.

### 2.1. Hereditary BMF

Inherited causes of BMF encompass a spectrum of genetic disorders that detrimentally affect the production of blood cells, originating from germline mutations, inherited from one’s parents, or occurring spontaneously (de novo). Predominantly, these inherited disorders follow an autosomal recessive inheritance pattern, including conditions such as Fanconi anemia, congenital dyserythropoietic anemias, Schwachman–Diamond syndrome, congenital megakaryocytic thrombocytopenia, and reticular dysgenesis. Conversely, a minor proportion of cases exhibits X-linked inheritance, as seen in sideroblastic anemia and dyskeratosis congenita, or an autosomal dominant pattern, exemplified by Diamond-Blackfan anemia.

#### 2.1.1. Fanconi Anemia (FA)

FA represents the most prevalent inherited form of BMF, with an incidence ranging from one to five cases per million, and a carrier frequency between 1 in 200 and 1 in 300. It arises from mutations in at least 20 distinct genes responsible for DNA damage repair, leading to genomic instability [6,7]. Clinically, FA is characterized by reduced blood cell counts, developmental anomalies, and an elevated cancer risk. Public health interventions focus on genetic counselling, bone marrow transplantation, and vigilant cancer surveillance.

#### 2.1.2. Diamond–Blackfan Anemia (DBA)

DBA is a notably rare congenital anemia defined by a failure in red blood cell (RBC) production (congenital erythroblastopenia), typically presenting within the first year of life, with symptoms such as fatigue, pale skin, macrocytic or normocytic anemia, and reticulocytopenia. The diagnosis of DBA can be complex, with some cases only identified in adulthood. Bone marrow aspiration and measurement of RBC adenosine deaminase (ADA) activity are crucial for diagnosis. Approximately 70–80% of DBA cases are linked to mutations in ribosomal protein genes, exhibiting an autosomal dominant inheritance pattern, particularly affecting *RPS19, RPL5*, *RPS26*, and *RPL11*. Mutations in non-ribosomal protein genes like *GATA1*, *TSR2*, and *HEATR3* have also been identified. The pathophysiology of DBA involves ribosomal stress, p53 activation, translational dysfunction, and abnormal inflammatory signaling pathways. Public health strategies include awareness campaigns and research into steroid therapy and bone marrow transplantation [8].

#### 2.1.3. Congenital Dyserythropoietic Anemia (CDA)

**Figure 2:**
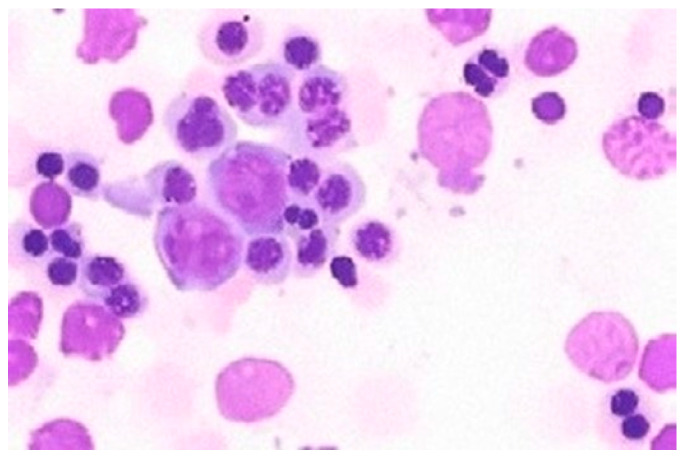
Bone marrow examination of a patient with CDA type II with multinucleated mature erythroblasts.

CDAs constitute a group of rare disorders marked by defects in RBC production due to genetic mutations impacting their development and erythropoietic maturation. CDAs are congenital and present from birth, characterized by ineffective erythropoiesis, with moderate to severe anemia and increased infection risk. The diagnosis is based on a bone marrow morphological examination characterized by abnormal erythroblastic features (Figure 2), hyperbilirubinemia leading to jaundice, and, potentially, splenomegaly. These conditions demonstrate genetic heterogeneity, with subtypes (CDA-I, CDA-II, and CDA-III, among others) each associated with specific genetic mutations affecting erythropoiesis. Treatment focuses on managing anemia and its symptoms, potentially including blood transfusions, iron store management, and bone marrow transplantation in severe cases [9].

#### 2.1.4. Sideroblastic Anemias (SAs)

**Figure 3:**
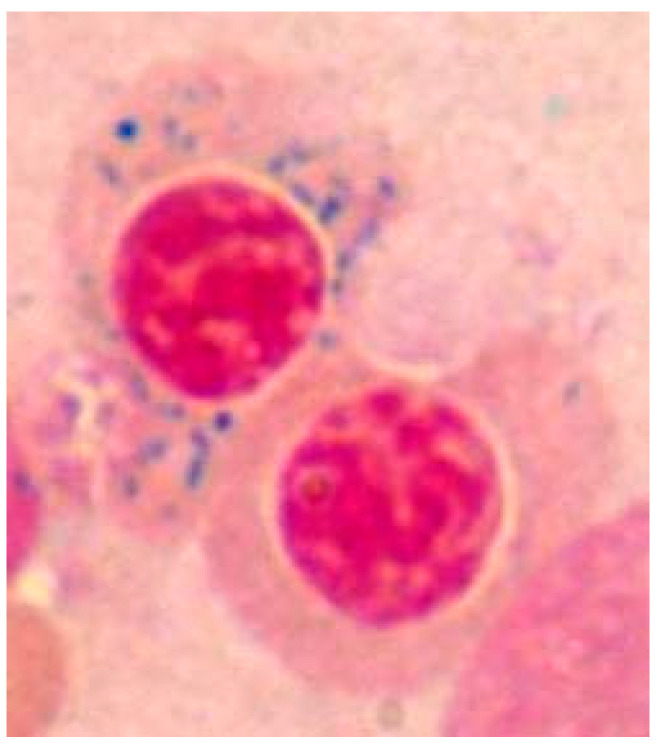
Typical sideroblast (erythroblast with hemosiderin surrounding the nucleus) observed after staining the bone marrow aspirate with Perls’ stain. This cell belongs to a patient with sideroblastic anemia.

SAs are characterized by pathological iron accumulation within the mitochondria of erythroid precursors called ring sideroblasts (Figure 3), resulting from mutations in the *ALAS2* and *ABCB7* genes on the X chromosome or mutations in genes on other chromosomes. Additional genetic disorders, such as Pearson syndrome or Wolfram syndrome, may also considered sideroblastic anemias [10].

### 2.2. Acquired BMF

Acquired BMF arises over time due to a multitude of factors, including autoimmune disorders, certain cancer types (such as large granular lymphocytic leukemia, lymphoma, and multiple myeloma), exposure to chemicals (including insecticides and pesticides), chemotherapy, other medications (such as antibiotics), rheumatoid arthritis, chronic kidney disease (CKD), myelodysplastic syndromes (MDS), acquired aplastic anemia (aAA), and paroxysmal nocturnal hemoglobinuria (PNH). Among these, acquired aplastic anemia (aAA) is the most encountered syndrome, characterized by fatigue, skin pallor, and an increased risk of infections and bleeding due to diminished peripheral blood cells. Public health initiatives are geared towards the establishment of bone marrow transplant donor registries and the exploration of therapies aimed at modulating the immune system. Notably, the pathogenesis of aAA is believed to be associated with the role of DNA telomere maintenance in hematopoiesis, where telomerase dysfunction may also contribute to inflammation and malignancy [11].

#### 2.2.1. Acquired Aplastic Anemia and Paroxysmal Nocturnal Hemoglobinuria

Acquired aplastic anemia (aAA) is a severe blood disorder marked by inadequate blood cell production in the bone marrow, affecting individuals of any age and gender. Unlike its congenital counterparts, acquired aplastic anemia develops later in life and can be triggered by immune attacks on the bone marrow, exposure to certain chemicals or drugs, radiation, viral infections, or other environmental agents [12]. Diagnosis involves blood tests and bone marrow biopsy to assess the blood cell levels and the health of blood-forming cells. Treatment options include immunosuppressive therapy, blood transfusions, and in critical cases, hematopoietic stem cell transplantation (HSCT), depending on the disease’s severity, the patient’s age, and overall health.

**Figure 4:**
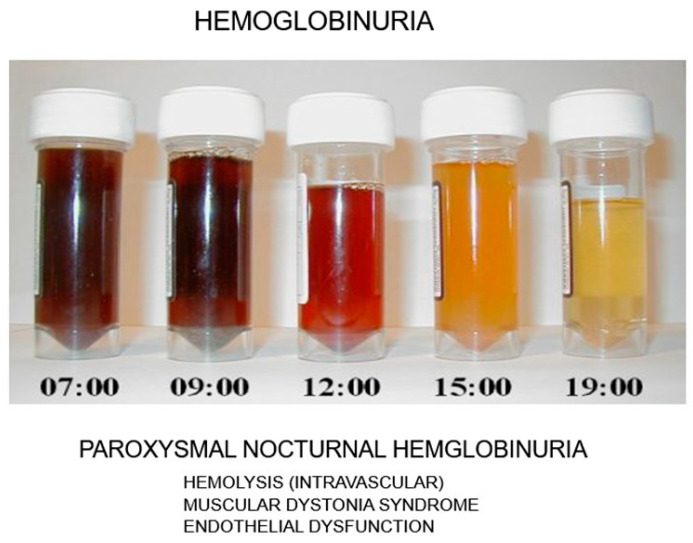
Urine taken at different times in the day from a patient with paroxysmal nocturnal hemoglobinuria (HPN). Dark urines appear in the early morning.

**Figure 5:**
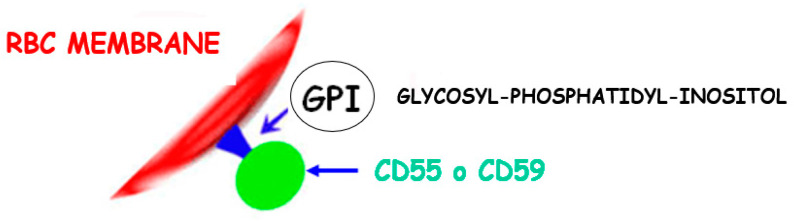
GPI anchor of proteins *CD55* and *CD59* to the RBC membrane.

More than 10% of patients with aAA may progress to develop clinically significant paroxysmal nocturnal hemoglobinuria (PNH), a rare and life-threatening disease due to the activation of plasma complement (C3). PNH symptoms include hemolysis; dark urine, especially in the early morning (Figure 4); and blood clot formation. Flow cytometric analysis often reveals a subclinical presence of PNH phenotype in granulocytes among aAA patients, indicating an expanded PNH clone possibly due to its resistance against autoimmune conditions. Interestingly, aAA is a known risk factor for PNH development, and, conversely, some individuals with PNH may also develop aAA, suggesting a complex interrelation between both disorders [13]. The disease typically affects all blood lineages (erythroid, megakaryocytic, and granulocyte/monocytic), primarily due to somatic mutations in the PIGA gene. This mutation leads to the loss of the glycosylphosphatidylinositol (GPI) anchor, a complex structure comprising a phosphoethanolamine linker, glycan core, and phospholipid tail that links the proteins *CD55* and *CD59* to the RBC membrane (Figure 5). In PNH, the lack of GPI results in the loss of *CD55* and *CD59* proteins, in unregulated plasma complement C3 activation and intravascular hemolysis. The expansion of a single clone of PIGA mutant, GPI-negative hematopoietic cells, plays a crucial role in the pathogenesis of PNH, though the mechanisms behind this clonal expansion remain largely unexplored. The overlap between aAA and PNH is well recognized, with immunosuppressive therapy showing to improve survival rates in severe aAA cases, further highlighting the intricate relationship between these hematologic disorders [14].

#### 2.2.2. Myelodysplastic Syndromes (MDS)

Like PNH, MDS involves a dysfunctional clone with a growth advantage over normal cells, and ribosomal defects like to those observed in DBA. MDS represents a heterogeneous group of myeloid abnormalities characterized by ineffective hematopoiesis, variable cytopenia’s, and a potential progression to acute myeloid leukemia (AML). The management of MDS presents various challenges, including anemia and diminished quality of life, prioritizing interventions to delay AML progression and improve survival in higher-risk cases. Despite advancements in molecular diagnostics enhancing the understanding of MDS pathogenesis, there remains a significant unmet need for effective and tolerable treatment strategies, particularly given the disease’s prevalence in older populations and its association with prior chemotherapy or radiation therapy exposure. Addressing these challenges necessitates a comprehensive approach, leveraging a deep understanding of MDS’s genetic landscape to develop personalized therapeutic strategies [15].

## 3. Rare Anemias Due to Red Blood Cell Defects

Red blood cell (RBC) defects can be classified into two categories: hereditary and acquired. Hereditary RBC defects are due to intrinsic structural or functional abnormalities in RBC components: hemoglobin (hemoglobinopathies), the cell membrane (membranopathies), and the enzymes of metabolism (enzymopathies). Acquired RBC defects are extrinsic to the RBCs, and result from abnormalities in blood plasma or the vascular system. In both categories, the consequence is a hemolytic syndrome characterized by anemia, of variable intensity, and a compensatory increase in bone marrow erythropoiesis reflected by an increased number of circulating reticulocytes (reticulocytosis).

### 3.1. Hereditary RBC Defects

Hereditary RBC defects are, in general, responsible for a decreased RBC lifespan in the circulation or hemolysis, associated with an increased erythroblastic regeneration and reticulocytosis. In general, the decrease in RBC lifespan led to a decrease in hemoglobin concentration and anemia (hemolytic anemia), but if the compensatory erythropoietic reaction can overcome the decrease of hemoglobin, the consequence is a compensated hemolysis without anemia and increased reticulocyte count. The three clinical hallmarks of the hemolytic syndrome, with or without anemia, are: 1. reticulocytosis, 2. splenomegaly, and 3. jaundice. This clinical phenotype is accompanied by increased serum bilirubin and lactate dehydrogenase (LDH), and decreased serum haptoglobin concentration. The inherited causes of RBC defects are classified as non-immune hemolytic anemias (NIHAs) and, as mentioned before, include hemoglobinopathies and thalassemias, membranopathies), and erythroenzymopathies [16].

#### 3.1.1. Hemoglobinopathies

**Figure 6:**
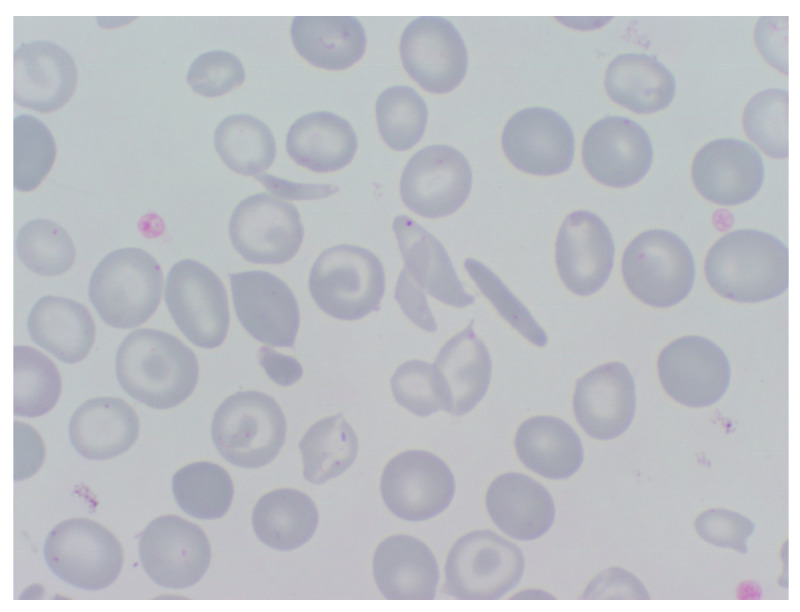
Sickle cell disease (SCD). Circulating sickle cells observed in an MGG-stained peripheral blood smear.

Hemoglobinopathies are genetic defects of the hemoglobin associated with chronic or acute anemia and other complications. They are classified into structural hemoglobinopathies and thalassemia’s. In Europe, hemoglobinopathies are still rare diseases, but their prevalence has significantly increased in many countries due to mobility and migration flows. The most frequent structural haemoglobinopathy is HbS (OMIM 603903), which results from a substitution of valine for glutamic acid in the sixth position of the globin beta chain in its homozygous form or combined with other hemoglobinopathies [17]. HbS is responsible for sickle cell disease (SCD), a clinical syndrome characterized by hemolytic anemia and severely painful Vaso-occlusive crises (VOCs). Both clinical manifestations are the consequence of the rigid misshapen RBCs (sickle cells) that appear after the decrease in HbS solubility triggered by hypoxia (Figure 6). VOC is due to multiple micro-infarcts and can occur in several tissues, including the long bones, pelvis, chest, and abdomen. The hemolytic crisis with anemia and the long-term pain due to VOC require frequent hospitalization and treatment by a multidisciplinary team. Another serious complication of SCD is acute chest syndrome (ACS), characterized by pulmonary infiltrates seen on chest radiography, often accompanied by fever and respiratory symptoms. SCD patients are also at a higher risk of infections due to functional asplenia and an altered immune response. All these adverse circumstances and the frequent hospitalization requirement are the cause of patients’ bad quality of life. SCD is very frequent in African populations due to the protection that it has offered against endemic malaria. During the last 30 years the migration impact of people from sub-Saharan Africa to Europe has created a growing health problem in the EU’s health services that has not yet been effectively addressed by member states (MS) authorities [18].

#### 3.1.2. Thalassemias

These are a group of genetic defects leading to a decrease in globin chain synthesis without structural hemoglobin involvement. Depending on their nature, thalassemias are classified into two main subtypes: β-thalassemia (decrease in β chains) and α-thalassemia (decrease in α chains). The Thalassemia International Federation (TIF), in collaboration with the World Health Organization (WHO), published a collection of independent reports, called “Global Thalassaemia Review 2022”, indicating that thalassemia occurs in 4.4 out of every 10,000 live births and is prevalent in Mediterranean coast geographical areas, Africa, the Middle East, southeast Asia, and southern China [19]. The report also highlights the huge heterogeneity and inequality that people affected by thalassemia still encounter in terms of access to quality healthcare services in many countries across the world. The global epidemiology of thalassemia is changing due to factors such as population genetics, improved survival rates, migration, and newborn screening programs. Moreover, the antenatal diagnosis of β-thalassemia has reduced its frequency in many Mediterranean countries. However, we must admit that a comprehensive understanding of the global prevalence, particularly with region- and subtype-specific estimates, is still limited.

#### 3.1.3. Membranopathies

**Figure 7:**
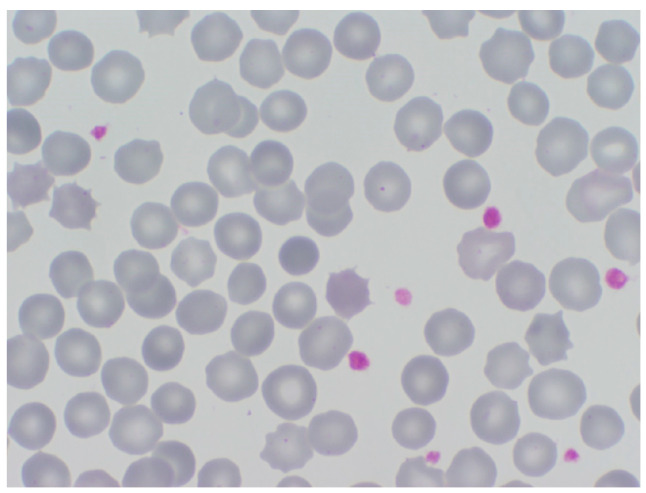
Stained peripheral blood smear from a patient with hereditary spherocytosis (HS). A typical spherocyte can be seen in the center of the picture.

These are a group of hereditary hemolytic anemias (HHAs) caused by quantitative or qualitative abnormalities in RBC membrane cytoskeleton proteins. They are mostly inherited in an autosomal dominant manner, but there is a relatively important percentage of cases transmitted in a recessive manner, without ruling out possible de novo mutations. The most frequently observed RBC membranopathies are hereditary spherocytosis (HS) and hereditary elliptocytosis (HE) caused by genetic defects of cytoskeleton proteins that disturb their interactions, impairing RBC deformability and leading to shortness of survival (hemolysis). HS is, by far, the most frequent cause of HHA, with a worldwide distribution and an estimated prevalence, in Europe, of about 1:2000 individuals. HS is easily diagnosed with a simple morphology examination on a stained blood smear when circulating spherocytes are evident (Figure 7). Genetic study has identified the most frequent mutations in HS to be SPTB (41%), followed by ANK1 (32%), SLC4A1 (18%), SPTA1 (4.5%), and EPB42 (4.5%). HE is less frequent than HS in Caucasians, but very frequent in the African population due to its relationship with endemic malaria. As in HS, the stained blood smear examination shows the presence of oval or elliptic RBCs and is the most useful diagnostic tool. A very rare membranopathy is hereditary xerocytosis (HX), due to a genetically abnormal membrane transport of cations (Na+ and K+) that leads to RBC dehydration. HX is characterized by slight anemia or compensated hemolysis, associated with marked reticulocytosis and increased MCHC (>350 g/L). HX, also known as dehydrated hereditary stomatocytosis (DHS), is caused by mutations in the genes PIEZO1 or KCNN4, which are responsible for encoding proteins that regulate the flow of ions in and out of RBCs [20]. The diagnosis of hereditary red blood cell (RBC) membranopathies continues to pose a clinical challenge. Comprehensive diagnostic approaches not only include the examination of RBC morphology and family history analyses but also benefit from the implementation of standard hemolysis tests. 

#### 3.1.4. Enzymopathies

**Figure 8:**
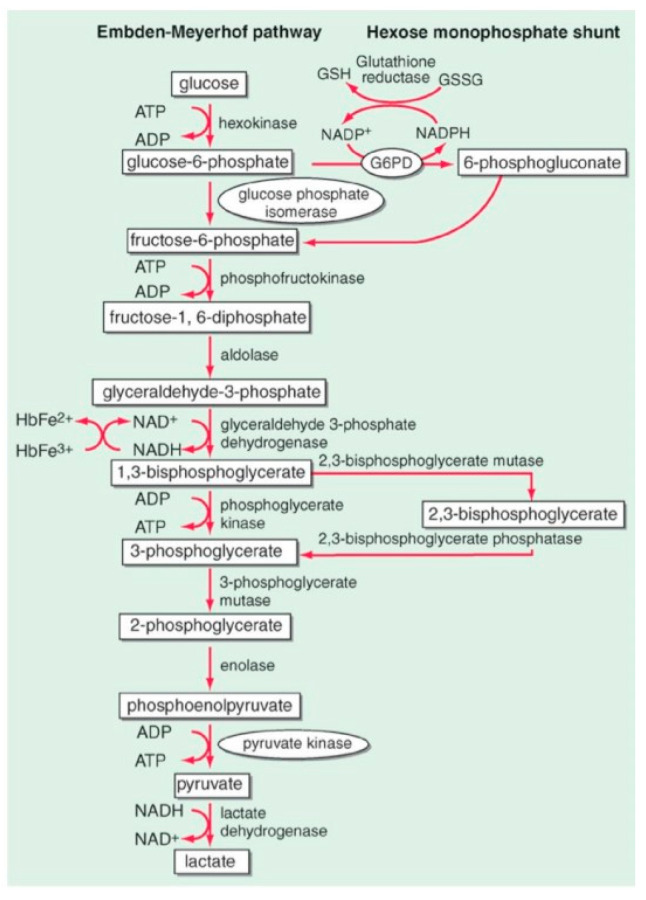
Glycolytic (Embden–Meyerhof pathway) and antioxidative metabolism (hexose–monophosphate pathway) are the two pillars of RBC metabolism.

**Figure 9:**
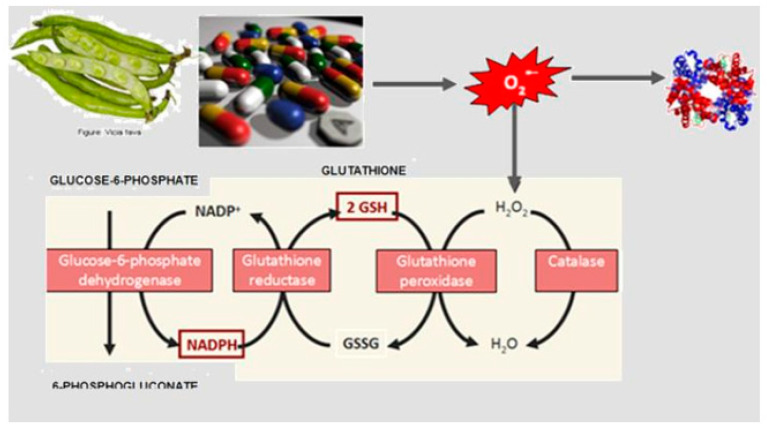
Oxidant threat with increase in H_2_O_2_ and the antioxidant effect of G6PD by producing NADPH necessary for maintaining the concentration of reduced glutathione (GSH).

These are a group of genetic disorders that affect the RBC metabolism (Figure 8). They are caused by mutations in genes that code for RBC enzymes, and at least 16 genetically determined conditions qualify as red blood cell enzymopathies [21]. RBC enzymopathies range in frequency from ultrarare to rare, except for glucose-6-phosphate dehydrogenase deficiency (G6PD), which is very common. G6PD is characterized by an acute hemolytic crisis after the exposure of RBCs to an oxidant trigger (drugs, fava beans, or infections). In G6PD deficiency, the antioxidant metabolism is impaired, and its metabolic consequence is an important decrease in reduced glutathione (GSH), the metabolite that protects RBC against an excess of peroxidation or oxidative treats (Figure 9). The diagnosis can be quite easy, such as when a child presents with dark urine after eating fava beans, or one of the drugs listed in Table 2. However, it can be quite difficult, such as when an adult presents with chronic mild anemia and gallstones. Very far in frequency from G6PD is pyruvate kinase deficiency (PKD), a rare genetic disorder caused by mutations in the PKLR gene, which codes for the RBC pyruvate kinase (PK) enzyme. Symptoms of PKD are those of chronic hemolytic syndrome with onset often in the neonatal period [22].

### 3.2. Acquired RBC Defects

Acquired RBC defects typically result from external insults to normal RBCs, such as the toxic effects of plasma components (autoantibodies, metals, or drugs), parasitic infections (e.g., malaria), or mechanical damage due to vascular abnormalities leading to intravascular RBC disruption (mechanical or microangiopathic hemolytic anemia).

#### 3.2.1. Autoimmune Hemolytic Anemia (AIHA)

Among the most prevalent acquired RBC defects is AIHA, which is mediated by antibodies and thus amenable to immunomodulatory treatments. A critical diagnostic test for distinguishing AIHA from non-immune hemolytic anemias (NIHAs) is the direct anti-human immunoglobulin test (DAT), also known as the Coombs test, which is positive in almost all cases of AIHA. The etiology of AIHA is often complex and obscured by its low prevalence, clinical heterogeneity, and associated complications. Both hereditary and acquired hemolytic anemias share common features, such as anemia, reticulocytosis, jaundice, and splenomegaly, complicating the identification of specific causes. This necessitates a combination of clinical expertise and specialized hematology laboratory support for accurate diagnosis. AIHAs are predominantly caused by warm autoantibodies (wAIHA) targeting IgG with or without complement C3. Initial treatment with corticosteroids yields a high response rate, but sustained remission is less common [23].

#### 3.2.2. Non-Immune Hemolytic Anemia (NIHA)

**Figure 10:**
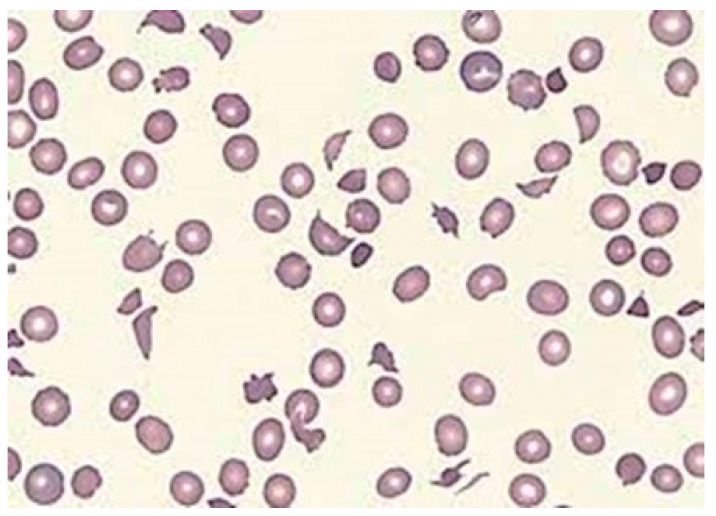
Typical fragmented RBCs (schistocytes) in a patient with atypical hemolytic uremic syndrome (aHUS).

On the other hand, NIHAs are attributed to a variety of plasma or vascular factors that compromise RBC structure or function. A notable example is microangiopathic hemolytic anemia (MAHA), characterized by RBC destruction within small blood vessels due to conditions such as thrombotic thrombocytopenic purpura (TTP), hemolytic uremic syndrome (HUS), malignant hypertension, and disseminated intravascular coagulation [24]. MAHA presents anemia and broken RBCs (schistocytes) on the blood film (Figure 10). Diagnosing MAHA involves a four-step process that includes recognizing chronic hemolytic anemia, excluding acquired causes, and ruling out hemoglobinopathies, membranopathies, or enzymopathies through DNA testing against an appropriate gene panel. While many MAHA patients respond well to supportive care, including blood transfusions, iron chelation, and splenectomy in selected cases, some exhibit severe extraerythrocytic manifestations challenging to manage. In the absence of such complications, MAHA may be amenable to hematopoietic stem cell transplantation (HSCT), gene therapy, or gene editing.

## 4. Rare Anemias Due to Iron Metabolism Defects

Hereditary iron metabolism defects are rare genetic disorders that represent a complex group of diseases affecting iron homeostasis that can lead to either iron overload or apparent iron deficiency [25]. Mitochondrial defects broaden the spectrum of these disorders, as mitochondria play a crucial role in cellular iron utilization, especially for heme and iron–sulfur cluster synthesis. Hereditary iron metabolic defects impact various body functions and organs, causing a range of health disorders, illustrating the intricate relationship between iron metabolism and mitochondrial function. From a clinical point of view, these disorders vary significantly in their presentation, ranging from asymptomatic conditions detected through routine screenings to severe disorders requiring lifelong management. Diagnosis typically involves genetic testing, blood tests to measure iron levels, and liver biopsy in some cases. Treatment strategies may include phlebotomy (blood removal) to reduce iron levels, iron supplementation, chelation therapy to remove excess iron, dietary modifications, and supportive care for associated symptoms. The management of these conditions often requires a multidisciplinary approach involving hematologists, geneticists, and other specialists.

Table 3 presents the genetic causes of these disorders, how they affect iron metabolism, and the main symptoms or effects associated with each disorder requiring a unique approach to diagnosis and management [26].

## 5. Diagnostic Tools for Rare Anemias: An Update

Diagnosing anemia requires a comprehensive evaluation of symptoms, blood tests, and the consideration of the individual’s clinical history. The generic nature of symptoms, such as fatigue and weakness, complicates the diagnostic process, demanding that healthcare professionals employ precision and timeliness in their interventions. Recent years have witnessed significant advancements in diagnostic assays and devices for rare anemias, heralding a new era of hope for precise diagnosis and efficacious treatments for affected individuals. These innovations aim to enhance diagnostic accuracy, expedite testing processes, and deepen the informational yield of diagnostic evaluations. Crucially, these advancements are pivotal in improving patient outcomes and formulating more effective treatment strategies. Among the modern diagnostic tools are automated blood cell analyzers and next-generation sequencing technologies. Additionally, the exploration of artificial neural networks to refine diagnostic precision underscores the progressive trajectory toward improved patient care for rare anemias, signifying a considerable advancement in diagnosis and management (Table 4).

### 5.1. Blood Testing and Point-of-Care Enhancement

Advances in blood testing have substantially improved the diagnosis and management of blood disorders, including rare anemias. Modern blood tests have evolved to offer unprecedented precision, sensitivity, and comprehensive data. Complete blood count (CBC) now provides intricate details on blood cell counts (RBCs, WBCs, and platelets), hemoglobin concentration, packed cell volume, and RBC indices (MCV, MCH, and MCHC). These enhanced parameters are instrumental in differentiating among various anemia types. In the context of hereditary hemolytic anemias, the focus has been on refining diagnostic tools and methodologies due to the complexity presented by abnormalities in erythrocyte structure, metabolism, and transport functions. The development of automated equipment’s leveraging of artificial neural networks represents a forward-looking approach in this domain [27].

The advent of smartphone applications and portable devices for anemia testing promises to extend diagnostic accessibility, especially in resource-constrained environments.

Recent advancements in the sophistication of POCT devices for rare anemias have significantly increased the efficiency, accuracy, and accessibility of anemia diagnosis and monitoring. This capability is essential for immediate diagnosis outside traditional laboratory settings and in acute scenarios or regions with limited laboratory access. Notable developments include the miniaturization of devices for on-site testing, novel biosensors, and bioanalytical techniques for enhanced analyte detection. Smartphone-based POCT devices, with their simplicity and affordability, are transforming mobile phones into potent diagnostic instruments, particularly beneficial in low-resource settings and for remote patient monitoring [28]. Furthermore, the integration of the Internet of Things (IoT), artificial intelligence (AI), and machine learning into POCT devices is bolstering their effectiveness and accuracy, facilitating real-time data analysis and enabling personalized medicine approaches.

### 5.2. Flow Cytometry Implementation

Flow cytometry has considerably advanced in its application for diagnosing hematologic conditions, including rare anemias. It has become a critical tool in hematology and oncology, propelled by enhancements in instrumentation and the broader availability of antibodies and fluorochromes. These improvements have led to more precise cell phenotyping and the identification of abnormal cell populations. In rare anemias, flow cytometry is particularly valuable for detecting abnormal RBC populations and assessing cell size and surface markers, essential for diagnosing conditions like hereditary spherocytosis (HS) and paroxysmal nocturnal hemoglobinuria (PNH).

For HS, flow cytometry is used to assess the integrity of band 3 and some cytoskeletal proteins. For this, the eosin-5-maleimide (EMA) binding test provides a quantitative measure of the reduction in band 3 and other membrane proteins critical for RBC integrity. The EMA binding test measures the capability of the fluorochrome EMA to bind band 3 that is decreased in HS due to the loss of membrane, offering a quick, reliable, and specific method for detecting HS.

**Figure 11:**
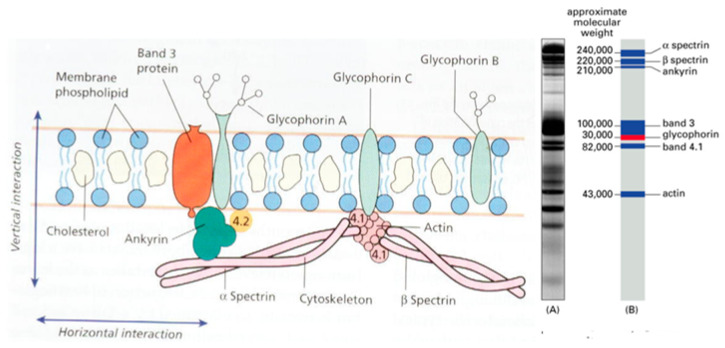
Electrophoresis of RBC membrane proteins using sodium dodecyl sulfate and polyacrylamide gel (SDS-PAGE). The proteins can interact in a horizontal and in a vertical sense.

For the diagnosis of HS, the EMA binding test can be complemented with the quantification of protein abnormalities that can be efficiently achieved through sodium dodecyl sulfate–polyacrylamide gel electrophoresis (SDS-PAGE) (Figure 11). SDS-PAGE evaluates RBC band 3 and other membrane proteins, providing an indirect measure of cytoskeletal health. Assessed alongside other methods, SDS-PAGE offers a unique insight into various congenital, and some acquired hemolytic anemias [29].

For PNH, flow cytometry allows for a precise and rapid analysis of the characteristics of thousands of cells in a sample of blood or bone marrow. Here, the cytofluorometer is used to detect the absence or reduced expression of GPI-anchored proteins on the surface of red blood cells, white blood cells, and platelets. A recent advance in the diagnosis and monitoring of PNH is the use of FLAER (fluorescent aerolysin), which binds specifically to the GPI anchor and detects GPI-deficient blood cells. The cells without GPI anchors (those affected by PNH) will not bind to FLAER and can be easily identified using flow cytometry. FLAER staining has significantly improved the diagnosis and monitoring of PNH by offering a highly sensitive and specific method for detecting cells deficient in GPI-anchored proteins, aiding in risk assessment, treatment decisions, and monitoring disease progression. Progress in flow cytometry has not only enhanced the diagnostic accuracy for conditions like PNH and HS but also facilitated the identification of therapeutic targets, significantly impacting the management and treatment of these disorders.

### 5.3. Red Blood Cell Physicochemical Properties

**Figure 12:**
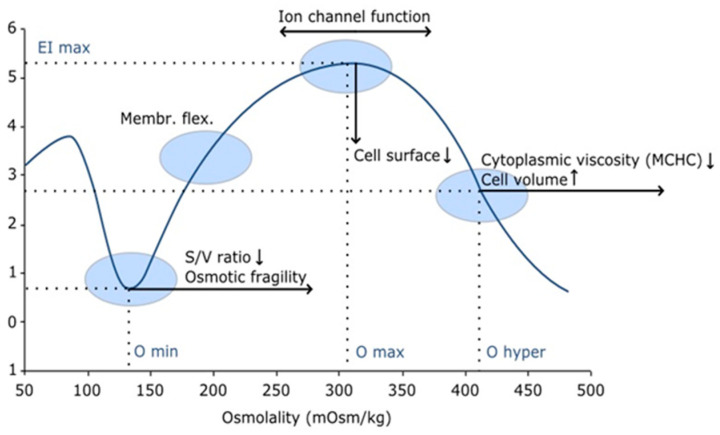
Lorrca osmoscan curve and the rheological parameters provided: EImax: RBC deformability; Omin: osmotic fragility; and Ohyper: RBC cytoplasmic viscosity.

Novel approaches based on RBC cell plasma interactions and deformability have been implemented in the last decade. Classical tests such as RBC osmotic fragility (OFT) and acidified glycerol lysis testing (AGLT) have been used for many years for the diagnosis of hereditary hemolytic anemias (HHAs), and specially for HS. Recently, the diagnostic landscape for hereditary hemolytic anemias (HHAs) has been enriched by the inclusion of red blood cell (RBC) deformability assessments, along with various other RBC rheological parameters. To this end, a specialized viscometer known as ektacytometer has been standardized under the name of “Laser Optical Rotational Red Cell Analyzer” (Lorrca^®^) (LoRRca MaxSis, Mechatronics, Hoorn, The Netherlands). This instrument employs an osmoscan module that precisely evaluates RBC deformability across an osmotic gradient. The technique, termed osmotic gradient ektacytometry (OGE), generates a profile that delineates the maximum elongation index (EImax), osmotic fragility (Omin), and RBC hydration status (Ohyper), as depicted in Figure 12. This advanced device proves immensely valuable in diagnosing hereditary spherocytosis (HS), and it is posited that the synergistic application of the EMA binding test (EBT), the OGE, and targeted next-generation sequencing (NGS) facilitates the diagnosis of nearly all membranopathies [30,31].

### 5.4. Proteomics and Biomarkers

Proteomics is a comprehensive study of proteins and their functions and has emerged as a crucial tool in the diagnosis of rare anemias. Through the analysis of protein expression profiles in blood and bone marrow samples, proteomics facilitates the identification of specific biomarkers and molecular pathways associated with various anemia forms. This approach is invaluable for rare anemias, where conventional diagnostic methods may fall short in specificity and sensitivity. The application of proteomics offers a nuanced understanding of disease mechanisms at the protein level, potentially enhancing diagnostic accuracy, predicting disease progression, and guiding the development of targeted therapies. As a result, proteomics has become a cornerstone in the diagnostic landscape for rare anemias, providing insights into disease mechanisms, enabling early detection, and laying the groundwork for personalized treatment strategies. Mass spectrometry (MS), a key technique in proteomic analysis, identifies and quantifies proteins in blood samples, including potential biomarkers for specific rare anemia types. Integrating proteomics with other omics approaches, such as genomics and metabolomics, yields a comprehensive view of rare anemias’ pathophysiology. These integrative analyses can uncover novel biomarkers and therapeutic targets, enhancing personalized medicine’s scope [32]. Proteomics sheds light on the pathophysiological mechanisms underlying rare anemias, enabling precise diagnoses. It also facilitates a personalized diagnosis approach by identifying individual protein expression variations, which are crucial due to the significant patient heterogeneity in rare anemias. Additionally, proteomic technologies can detect early, subtle changes in protein expression, allowing for earlier disease diagnosis and progression monitoring. In the realm of RBCs, proteomics has provided insights into cellular metabolism and changes occurring during RBC storage for transfusion, vital for improving certain anemias’ safety. Mass spectrometry’s sensitivity enables the detection of protein expression changes, post-translational modifications, and abnormal proteins presence.

### 5.5. Molecular Genetics

Genetic testing plays a pivotal role in diagnosing rare anemias, especially in hereditary hemolytic anemias (HHAs), thalassemia syndromes, and sickle cell disease (SCD), offering definitive diagnoses essential for early detection and management. Recent advancements in genetic testing, including third-generation sequencing techniques, have enhanced our understanding and diagnosis of rare anemias by targeting known gene variations [33]. Moreover, comprehensive genetic testing is crucial for diagnosing acquired bone marrow failure (BMF) syndromes, aiding in the identification of specific genetic abnormalities without necessitating immediate therapy. Techniques like quantitative PCR (qPCR) and next-generation sequencing (NGS) have revolutionized rare anemias diagnosis by enabling rapid, sensitive analysis of DNA or RNA sequences, identifying mutations, and providing insights into genetic profiles.

#### 5.5.1. Quantitative PCR (qPCR)

Also known as real-time PCR, qPCR is a highly sensitive and specific method used in molecular biology for the quantification of DNA or RNA sequences. In some rare anemias, when the issue might not be a mutation in the DNA, but rather abnormal levels of gene expression, qPCR allows for the quantification of specific gene transcripts, helping in the diagnosis of such conditions; genetic counselling; and for monitoring the disease progression and the effectiveness of treatment by quantifying changes in relevant genetic markers over time [34].

#### 5.5.2. Next-Generation Sequencing (NGS)

NGS has revolutionized the diagnosis of rare anemias. It enables the rapid sequencing of large segments of DNA and allows for the rapid and comprehensive analysis of multiple genes associated with these conditions. This technique can identify both well-known and novel mutations, providing a detailed genetic profile of the patient [35]. It is particularly useful in diagnosing conditions with a genetic basis, such as hereditary spherocytosis, thalassemia’s, and sickle cell disease, where multiple genetic mutations may be involved. Different approaches to this molecular testing include custom-designed targeting panels (t-NGS), whole-exome sequencing (WES), or whole-genome sequencing (WGS) [36].

For certain types of rare anemias, such as thalassemia syndromes and sickle cell disease, genetic testing can provide a definitive diagnosis. This is especially important for early detection and diagnostic strategies that are crucial for the effective management and treatment, offering more personalized and accurate medical care for patients with these conditions [37]. One notable development is the use of third-generation sequencing techniques for the analysis of rare genetic variants associated with thalassemia. This approach involves long PCR-based sequencing, which targets known structural variations, single-nucleotide variations (SNVs), and insertions and deletions (indels) in key genes like *HBA1*, *HBA2*, and *HBB*. This method has been instrumental in identifying rare clinically significant SNVs, enhancing the understanding and diagnosis of various forms of thalassemia [38].

Also, for acquired rare anemias due to BMF, the incorporation of comprehensive genetic testing is especially important in patients not requiring immediate therapy. Accordingly, the detection of acquired 6p copy number-neutral loss of heterozygosity using bone marrow array for the diagnosis of aAA and the measure of telomere length by flow-FISH for short telomere syndromes and other inherited BMF are important advances in the diagnosis of BMF syndromes [5].

### 5.6. Artificial Intelligence (AI) and Machine Learning

AI and machine learning have significantly advanced rare anemia diagnosis by analyzing large datasets to identify patterns and markers associated with these conditions [39]. From machine learning algorithms analyzing patient data to deep learning models evaluating blood smears and bone marrow biopsies, AI enhances diagnostic accuracy, predicts treatment outcomes, and facilitates personalized medicine. Below are some of the recent applications of AI in diagnosing rare anemias.

#### 5.6.1. Machine Learning Algorithms for Data Analysis

Machine learning algorithms have been used to analyze patient data, including genetic information, to identify markers associated with rare anemias. This can help in diagnosing conditions like Diamond–Blackfan anemia (DBA) or sideroblastic anemia, which may not be easily identifiable through traditional methods. Deep learning models for image analysis have been applied to the analysis of blood smears and bone marrow biopsies. These models can identify subtle abnormalities in cell morphology that are indicative of rare anemias, improving the accuracy of diagnoses.

#### 5.6.2. Predictive Analytics for Treatment Outcomes

AI models are used to predict how patients with rare anemias might respond to different treatments. This is particularly useful for tailoring individualized treatment plans and for the management of diseases with complex treatment regimens.

#### 5.6.3. Genetic Data Analysis

Used to analyze and identify mutations associated with rare anemias. This not only aids in diagnosis but also helps in understanding the disease’s pathophysiology, which can lead to the development of targeted therapies. 

#### 5.6.4. AI’s Integration with Electronic Health Records (EHRs)

Integration also has applications in genetic data analysis and patient monitoring. These integrated AI systems are transforming the rare anemia diagnostic and management landscape by providing clinicians with real-time decision support. Moreover, they can help in the early detection of rare anemias, potentially leading to earlier intervention and better patient outcomes. 

#### 5.6.5. Patient Monitoring and Remote Diagnostics

Along with AI-enabled tools, these are also being used for continuous monitoring of patients with rare anemias. These tools can alert healthcare providers to changes in a patient’s condition that may require intervention, facilitating more proactive disease management.

#### 5.6.6. Collaborative Research Platforms

AI can facilitate collaborative research by enabling the sharing and analysis of clinical data from patients with rare anemias across different institutions. This collective approach is crucial for rare diseases, where individual institutions may not encounter enough cases to draw significant conclusions [40].

### 5.7. Telemedicine (TM) and Remote Patient Monitoring (RPM)

TM and RPM have seen substantial growth, particularly after the COVID-19 pandemic, offering patients with RAs access to expert consultations and clinical care remotely. RPM has proven to be a powerful tool, especially in the management of chronic and acute conditions from the patient’s home. This not only reduces travel costs and the risk of infections for patients, but also allows for more effective management of various conditions such as hypertension, diabetes, and heart disease. Moreover, patients with rare anemias can receive expert consultations and follow-up clinical care remotely, especially beneficial for patients living in areas with limited access to specialized healthcare facilities. Despite their many benefits, both TM and RPM face challenges. These include the digital divide, where technology, digital literacy, and broadband internet access are major barriers. Moreover, despite these benefits, there are uncertainties regarding reimbursement for these services. Hopefully, medical associations and policymakers are working to address these barriers [41].

### 5.8. Clinical Decision Support Systems (CDSSs)

CDSSs are evolving to integrate sophisticated algorithms and diverse data sources, providing clinicians with evidence-based guidance for more accurate and efficient decision-making. These systems, integrated with EHRs, leverage a knowledge base, an inference engine, and a communication mechanism to offer everything from drug interaction information to diagnostic support and therapy planning, emphasizing the importance of user-friendly design for effective clinical application. The communication mechanism allows for the system to present information in a friendly user format, and the functions of CDSS can range from offering basic information, such as drug interactions and guidelines, to more advanced functions like diagnostic support, therapy planning, and prognosis prediction. The design of the user interface in a CDSS is crucial and needs to be intuitive to ensure that healthcare providers can access and understand the information and recommendations provided by the system effectively [40].

## 6. Current Treatments for Rare Anemias: A Revolution

The treatment landscape for rare anemias, encompassing both hereditary and acquired forms, is rapidly evolving with notable progress in gene therapy, targeted medications, and supportive care. These developments hold promise for enhancing outcomes and quality of life for patients afflicted with these conditions. However, challenges related to accessibility, cost, and long-term safety persist, necessitating careful consideration for the future. The evolution of treatment strategies for rare anemias underscores the transformative potential of personalized medicine, driven by advancements in gene therapy and precision medicine. These emerging strategies hold promise for revolutionizing the management and outcomes of rare anemias, marking a significant step forward in this field of hematology Moreover, it signifies a transition from conventional supportive therapies to interventions with disease-modifying potential, including the integration of gene therapy [42].

A crucial requirement for managing rare anemias effectively lies in adopting a patient-tailored approach. This approach is indispensable for delivering care that is both efficacious and safe, thereby enhancing patient outcomes and improving their quality of life. Key components of this approach encompass considerations of individual characteristics and the variability in drug response. Recent strides in treating rare anemias, whether congenital or acquired, encompass advancements in managing hemoglobinopathies, hereditary hemolytic anemias (HHAs), autoimmune hemolytic anemia (AIHA), bone marrow failure (BMF), and paroxysmal nocturnal hemoglobinuria (PNH). The emergence of novel strategies for treating rare anemias is underpinned by an enhanced comprehension of the underlying pathophysiology of these conditions.

### 6.1. Hereditary Rare Anemias

Since congenital anemias encompass a range of disorders, including, but not limited to, sickle cell disease, thalassemia, Fanconi anemia, and Diamond–Blackfan anemia, emerging strategies can vary widely in their approach, from gene therapy to novel pharmaceuticals and bone marrow transplantation enhancements. The emerging strategies for the treatment of rare congenital anemias are summarized in Table 5, where an overview and simplification of the complex and rapidly evolving treatment landscape regarding the different types of anemia is provided. This includes their targets, approach to the mechanism of the treatment, and status of clinical development. The clinical development stage can vary significantly within each category, with some treatments being at the forefront of research and others already in clinical use. Additionally, “Key Considerations” reflect both the potential benefits and the challenges or limitations associated with each strategy, including, but not limited to, safety concerns, accessibility, and the need for long-term studies to fully understand the implications of these emerging treatments. For this, the following strategies have been considered: 1. gene therapy, 2. targeted drug treatments, 3. advances in iron chelation therapy, and 4. stem cell transplantation and 5. treatment for acquired rare anemias.

#### 6.1.1. Gene Therapy

Gene therapy represents a paradigm shift in the treatment landscape of hereditary anemias, promising a potential cure, particularly in diseases such as beta-thalassemia and sickle cell disease. The shift from traditional symptomatic and supportive treatments to gene therapy holds significant promise. Ongoing research and clinical trials in this domain are anticipated to yield further enhancements, rendering these therapies more accessible and safer in the future [43]. These are the following: 1. Gene addition therapy, utilizing lentiviral vectors for gene addition therapy presents a promising avenue for addressing beta-thalassemia. This methodology involves genetically modifying autologous CD34+ cells with a functional beta-globin gene delivered via lentiviral vectors. 2. Clinical trials such as Lentiglobin BB305, GLOBE, and TNS9.3.55 aim to evaluate the efficacy and safety of this approach, offering prospects for a curative option for individuals with transfusion-dependent beta-thalassemia (TDBT) [44]. 3. CRISPR-Cas9 technology is a revolutionary genome editing tool that facilitates precise modifications to DNA sequences. It functions by using the Cas9 enzyme and a guide RNA to target specific DNA sequences, enabling the addition, removal, or alteration of genetic material. CRISPR-Cas9 application in gene therapy holds vast potential, particularly in the treatment and prevention of genetic diseases such as sickle cell disease, beta-thalassemia, and hemophilia. However, further research is warranted to ensure its safety and effectiveness, with ethical considerations surrounding its use in human germline cells [45]. 4. Reactivate fetal hemoglobin (HbF) production emerges as a promising approach in treating blood disorders like sickle cell disease (SCD). This strategy aims to reduce disease complications and enhance the quality of life by inhibiting the sickling process in red blood cells. The gene therapy approach focuses on reactivating the production of fetal hemoglobin (HbF) in adult cells by suppressing the BCL11A gene, a silencer of HbF production. Clinical trials such as the Gene Transfer Study Inducing HbF in SCD trial (GRASP) evaluate the efficacy and safety of these techniques, with recent approvals marking significant milestones in gene therapy for blood disorders [46]. 5. The correction of ribosomal protein gene mutations has been facilitated by the advances in genetic engineering and gene therapy and offers promising prospects for correcting ribosomal protein gene mutations associated with disorders like Diamond–Blackfan anemia (DBA). Techniques such as CRISPR/Cas9 genome editing enable targeted interventions at the genetic level, holding potential for repairing or replacing faulty genes and improving treatment outcomes [8,47]. The precise approach depends on the specific mutation and the disorder being treated, with ongoing research focusing on improving the efficacy and safety of these interventions. Precision diagnosis and tailored treatment for BMF is a recent approach for treatment that emphasizes precise diagnosis and tailored treatment based on genetic factors. Accurate diagnosis is paramount for determining the appropriate treatment strategy and surveillance, with ongoing research exploring gene editing as a potentially curative approach. Advances in genomic technologies have been instrumental in identifying new BMF syndromes, underscoring the importance of ongoing research to refine treatment techniques and improve outcomes [48,49]. Non-ribosomal protein gene mutations, such as GATA1, TSR2, and HEATR3, associated with DBA, and their respective inheritance patterns seem to also have clinical implications.

#### 6.1.2. Targeted Drug Treatments

These represent a notable advancement in the treatment of some RAs. An example is the FDA approval of Mitapivat (Pyrukynd, Agios Pharmaceuticals, Inc.©, Cambridge, MA, USA) for the treatment of hemolytic anemia in adults that is the first FDA-approved disease-modifying therapy for PK deficiency. Mitapivat efficacy underwent evaluation through two studies conducted on patients with PK deficiency (PKD): a randomized, placebo-controlled clinical study and a single-arm study. In the randomized study, 40% of participants treated with Mitapivat exhibited a significant increase in hemoglobin concentration, whereas in the single-arm study, 33% of participants experienced a reduced need for blood transfusions, with 22% requiring no transfusions during the final 24 weeks of the study period. These findings underscore Mitapivat’s potential as a promising novel treatment option for PK deficiency [49]. Recent non-gene therapy drug treatments for thalassemia and sickle cell disease have witnessed significant progress, underscoring the continual evolution of treatment strategies that target specific facets of these blood disorders. Luspatercept, an activin receptor ligand trap, represents the first erythroid maturation agent approved for adult patients with transfusion-dependent β-thalassemia [50].

For SCD, novel pharmacological options have emerged to complement the established use of hydroxyurea. These include L-glutamine, Voxelotor, and crizanlizumab, all of which have received approval for SCD and target various aspects of the disease’s pathophysiology. These drugs have demonstrated efficacy in elevating hemoglobin levels and reducing markers of hemolysis. L-glutamine, an amino acid supplement approved for patients older than 5 years, offers a new avenue for managing clinical complications associated with SCD. Voxelotor, approved for patients aged 12 years and older, aids in mitigating hemoglobin S polymerization, a critical concern in sickle cell disease pathophysiology. Crizanlizumab, a monoclonal antibody approved for patients aged 16 years and older, addresses Vaso-occlusion, a common complication in SCD. Furthermore, the introduction of Mitapivat and Etavopivat, selective activators of RBC pyruvate kinase (PK), adds to the array of treatment options available. It is imperative to acknowledge that the advancement of these treatments and diagnostic tools also underscores the necessity for equitable practices in funding and accessibility. Ensuring that patients from diverse backgrounds and socioeconomic statuses have access to these innovations is essential for fostering inclusive progress in rare disease research and treatment [51].

#### 6.1.3. Recent Advancements in Iron Chelation Therapy

Iron chelation has markedly enhanced the management and prognosis of patients with conditions associated with iron overload. These advancements, along with evaluations of existing iron chelating therapies, emphasize their efficacy, safety profiles, and the critical role of treatment adherence in achieving optimal outcomes for patients with conditions such as sickle cell disease (SCD), thalassemia, and congenital dyserythropoietic anemia (CDA), offering promising avenues for improved management of iron overload and its complications. Key developments: The landscape of iron chelation, has been enriched by several new and improved chelators, such as deferasirox, deferiprone, and deferoxamine, that have rendered the therapy more effective and convenient, particularly with the availability of oral formulations, thus enhancing patient compliance [52]. Moreover, the approach to iron chelation has become increasingly personalized by tailored chelating strategies that use advancements in tissue iron quantification. This enables therapies to be tailored to individual patient needs, aiming for precise control of iron levels and consequent reduction in associated morbidity and mortality [53]. Finally, a significant step forward has been the development of new oral formulations, such as the film-coated tablet (FCT) version of deferasirox. These formulations not only enhance the bioavailability of chelating agents but also aim to mitigate side effects, thereby improving treatment tolerability for patients [54].

#### 6.1.4. Hematopoietic Stem Cell Transplantation (HSCT)

Advances in HSCT for rare anemias have been centered on enhancing outcomes and comprehending the long-term effects and challenges associated with these treatments. Key developments include innovations in HSCT for Fanconi anemia (FA) that have delved into analyzing long-term survival, organ function, malignancy risks, and psychosocial adjustment following HSCT in FA patients. These studies offer valuable insights into the efficacy and safety of HSCT for FA treatment, emphasizing the significance of meticulous patient selection and management strategies to optimize outcomes [55]. Research into outcome predictors for HSCT in FA patients has encompassed analyses of patient and transplant characteristics such as age, disease status (e.g., presence of associated MDS or acute myeloid leukemia (AML)), and specifics of the conditioning regimen. These endeavors aim to refine HSCT protocols and improve survival rates and quality of life for transplant recipients [56]. Recent advances in the broader field of hematopoietic stem cell biology (SCB) have allowed for significant innovations, with implications for the treatment of RA and other hematologic conditions. Advances in understanding molecular mechanisms of stem cell differentiation and the development of novel techniques for stem cell manipulation and tracking hold potential to enhance the efficacy and safety of stem cell therapies. These innovations include the discovery of specific enzyme roles in embryonic development, utilization of single-cell and spatial transcriptomics to elucidate clonal relations in cell populations, and insights into the timing of functional hematopoietic stem and progenitor cell activity during fetal development [57]. These recent advances underscore the rapid progress in the field of HSCT and SCB, offering new hope for patients with RA and other hematologic diseases. The focus on improving long-term outcomes, understanding the molecular underpinnings of stem cell behavior, and refining transplantation techniques is poised to significantly impact the treatment landscape for RAs. Finally, regenerative medicine and stem cell research are pivotal, not only in understanding and diagnosing rare anemias, but also in developing innovative, effective, and personalized treatment options. Ongoing advancements in these domains hold great promise for improving the lives of patients facing these challenging conditions.

### 6.2. Acquired Rare Anemias

For acquired rare anemias, treatment strategies can be quite different from those for congenital anemias, focusing more on addressing the underlying cause, which could be autoimmune diseases, infections, exposure to toxic substances, or other secondary factors. Table 6 summarizes the emerging strategies for the treatment of acquired rare anemias and highlights the diversity in approaches being explored to manage and treat acquired rare anemias, which often require a multifaceted strategy involving both direct treatment of the anemia and addressing its root cause. The clinical development stage varies widely, with some strategies already integrated into clinical practice for certain conditions, while others are still under investigation in clinical trials. Key considerations include the balance between efficacy and potential side effects, the need for individualized treatment plans based on the underlying cause and patient condition, and the economic impact of long-term treatments, especially for those requiring novel or expensive therapies.

#### 6.2.1. Acquired Aplastic Anemia (aAA) and Pure Red Cell Aplasia (PRCA)

For aAA and PRCA strategies typically involve immunosuppression with corticosteroids, often in combination with cyclosporine A [58]. Recent studies have emphasized the potential benefits of incorporating eltrombopag and sirolimus, particularly in refractory cases or those intolerant to cyclosporine A. Eltrombopag, either as monotherapy or in combination with other therapies, has shown promising improvements in patient outcomes. However, its specific role in Diamond–Blackfan anemia (DBA) warrants further clinical investigation and validation [59].

#### 6.2.2. Autoimmune Hemolytic Anemia (AIHA)

For AIHA, therapeutic approaches vary based on the subtype, such as warm autoimmune hemolytic anemia (wAIHA) and cold agglutinin disease (CAD). For those who do not respond to corticosteroids, rituximab offers complete remission in a significant portion of patients, with some achieving long-term remission. Nevertheless, a substantial number of patients do not respond to rituximab and may require treatment with erythropoiesis-stimulating agents or immunosuppressive drugs. In refractory cases, splenectomy may provide long-term remission for most patients [60].

Emerging treatments for wAIHA, such as fostamatinib, rilzabrutinib, and FcRn inhibitors, are promising, leading to the proposal of a new treatment algorithm aimed at minimizing corticosteroid use. Recent insights include the utilization of complement inhibiting antibodies and plasmapheresis as effective interventions. Moreover, targeted therapies beyond rituximab are emerging for wAIHA and CAD, including B-cell/plasma cell targeting agents, complement inhibitors, and inhibitors of extravascular hemolysis, offering additional treatment options.

#### 6.2.3. Paroxysmal Nocturnal Hemoglobinuria (PNH)

PNH treatment has evolved from intravenous anti-C5 eculizumab to newer complement inhibitors like ravulizumab and pegcetacoplan. These advancements provide diverse options for PNH management, targeting different components of the complement system to reduce red blood cell destruction [61]. Moreover, they underscore a shift towards more personalized and targeted approaches in rare anemia treatment, moving beyond traditional supportive care to embrace disease-modifying agents and cutting-edge gene therapy techniques [62]. Precision medicine now plays a crucial role in tailoring treatment strategies to individual patients. Addressing the complexities of anemia requires a multifaceted approach involving public awareness, improved diagnostics, targeted treatments, and ongoing research endeavors. By understanding the nuances of anemia and fostering collaboration across healthcare sectors, we can strive towards mitigating its challenges and improving the lives of those affected by these conditions. Opportunities for progress lie in holistic strategies, interdisciplinary collaboration, and international efforts to alleviate the burden of rare anemias worldwide.

## Figures and Tables

**Table 1:** General classification of rare anemias.

Hereditary (>80%) Erythropoietic defects (non-regenerative anemias) Fanconi anemia (FA) Diamond–Blackfan anemia (DBA) Congenital dyserythropoietic anemia (CDA) RBC defects (regenerative anemias) Thalassemia syndromes (Cooley anemia) Sickle cell disease (SCD) Hereditary membranopathies ○ Hereditary spherocytosis (HS) ○ Hereditary elliptocytosis (HE) ○ Hereditary stomatocytosis (HSt) Erythroenzymopathies ○ Glucose-6-phosphate dehydrogenase deficiency (Favism) ○ Pyruvate kinase deficiency (PKD) ○ Ultra-rare erythroenzymopathies associated or not with muscular or neurological disease Iron metabolism defects (non-regenerative anemias) Congenital sideroblastic anemia (CSA) Non-sideroblastic anemias with microcytosis (IRIDA) * Acquired (<20%) Erythropoietic defects (non-regenerative anemias) Bone marrow aplasia (BMA) Pure red cell aplasia (PRCA) Myelodysplastic syndromes (MDS) RBC defects (regenerative anemia) Paroxysmal nocturnal hemoglobinuria (HPN) Blood plasma abnormalities (regenerative anemias) Autoimmune hemolytic anemia (AIHA) Microcirculation defects (regenerative anemias) Hemolytic uremic syndrome (HUS)

* IRIDA: Iron-refractory iron deficiency anemia.

**Table 2:** Common drugs to be avoided or used with caution in G6PD deficiency.

Acetaminophen	Primaquine
Acetylsalicylic acid	Rasburicase
Chloramphenicol	Streptomycin
Chloroquine	Sulfacetamide
Colchicine	Sulfanilamide
Diamino diphenyl sulfone	Sulphapyridine
Diphenhydramine	Sulfacytine
Glyburide	Sulfadiazine
Isoniazid	Sulphaguanidine
L-Dopa	Sulfamethoxazole
Methylene blue	Sulfisoxazole
Nitrofurantoin	Trimethoprim
Phenazopyridine	Tripelennamine
Vitamin C	Fava bean extract

**Table 3:** Key aspects of rare hereditary iron metabolism disorders, including those with mitochondrial involvement.

Disorder	Genetic Cause	Primary Effect on Iron Metabolism	Main Symptoms/Effects
Hereditary hemochromatosis (HH)	Mutations in *HFE*, *HJV*, *HAMP*, *TFR2*, *SLC40A1* genes	Iron overload due to increased absorption	Liver disease, diabetes, heart abnormalities, joint pain
Mitochondrial lactic acidosis and sideroblastic anemia (MLASA)	Mutations in *PUS1*, *YARS2* genes	Impaired mitochondrial protein synthesis leading to sideroblastic anemia	Muscle weakness, lactic acidosis, sideroblastic anemia
Aceruloplasminemia	Mutations in the *CP* gene	Iron accumulation in organs due to lack of ceruloplasmin	Neurological symptoms, retinal degeneration, diabetes, anemia
X-linked sideroblastic anemia	Mutations in the *ALAS2* gene	Impaired heme synthesis leading to mitochondrial iron overload in erythroblasts	Anemia, iron overload in liver
Pearson syndrome	Deletions in mitochondrial DNA	Mitochondrial dysfunction affecting erythropoiesis and other systems	Sideroblastic anemia, pancreatic dysfunction, failure to thrive, metabolic acidosis
Atransferrinemia	Probable mutation of TF gene	Lack of transferrin leading to iron overload and deficiency	Iron overload in organs, severe iron deficiency anemia
Ferroportin disease	Mutations in the *SLC40A1* gene	Altered iron export from cells leading to iron accumulation and anemia	Iron overload, particularly in liver, alongside anemia
Iron-refractory iron deficiency anemia (IRIDA)	Mutations in the *TMPRSS6* gene	Overproduction of hepcidin leading to impaired iron absorption	Iron deficiency anemia resistant to oral iron supplements

**Table 4:** Enhanced diagnostic tools for rare anemias.

Blood testing enhancements	Modern automated hematology analyzers offer great precision, sensitivity, and comprehensive results of essential hematologic parameters, aiding in differentiating among various anemia types.
Point-of-care testing (POCT) developments	POCT devices have evolved to facilitate rapid and accurate hemoglobin measurements and other data outside traditional laboratory settings, with notable technological enhancements.
Flow cytometry advances	Improvements in flow cytometry equipment and available antibodies have led to more precise cell phenotyping and identification of abnormal cell populations in rare anemias such as membranopathies and PNH.
Red Blood Cell Physicochemical Properties	Novel approaches based on RBC cell membrane and plasma interactions Improvement of RBC deformability measurement by Osmotic Gradient Ektacytometry (OGE)
Proteomics and biomarkers	The comprehensive study of proteins and their functions (proteomics) has become crucial in diagnosing rare anemias by identifying specific biomarkers and molecular pathways.
Molecular genetics	Advancements in genetic testing, including third-generation sequencing techniques, have improved the diagnosis of rare anemias by targeting known gene variations.
Artificial intelligence and machine learning	These technologies have advanced the diagnosis of rare anemias by analyzing large datasets to identify patterns and markers associated with these conditions.
Telemedicine (TM) and remote patient monitoring (RPM)	These offer patients with RAs access to expert consultations and clinical care remotely.
Clinical decision support systems (CDSSs)	These integrate sophisticated algorithms and diverse data sources, providing clinicians with evidence-based guidance for more accurate and efficient decision-making.

**Table 5:** Emerging strategies for the treatment of rare hereditary anemias.

Disease	Treatment Strategy	Mechanism/Approach	Clinical Development Stage	Key Considerations
Sickle cell disease	Gene therapy	Correction or modification of the HBB gene encoding beta-globin	Clinical trials	Potential for a cure; high cost; accessibility issues
Thalassemia	Gene editing (CRISPR/Cas9)	Editing the HBB gene or its regulators to increase fetal hemoglobin production	Clinical trials	Off-target effects; long-term efficacy and safety
Fanconi anemia (FA)	Gene therapy	Delivery of a functional copy of the faulty gene (e.g., FANCA)	Early research/preclinical	Limited by vector capacity; potential for immune responses
Diamond–Blackfan anemia (DBA)	Corticosteroids and stem cell transplantation	Corticosteroids to increase red blood cell count, Bone marrow transplant (BMT) for severe cases	Standard treatment	Steroid side effects; risk of graft-versus-host disease
Pyruvate kinase deficiency (PKD)	Small molecule activators (Mitapivat)	Activation of pyruvate kinase to improve red blood cell metabolism	Approved treatment	Specificity and side effects; long-term impact on metabolism
Congenital Dyserythropoietic Anemia (CDA)	Iron chelation and gene therapy	Management of iron overload; correction of the genetic defect	Iron chelation in use; gene therapy in preclinical	Iron chelation side effects; gene therapy’s future potential
Hereditary spherocytosis (HS)	Splenectomy and erythropoiesis-stimulating agents	Removal of the spleen to reduce hemolysis; agents to increase red blood cell production	Standard and emerging	Splenectomy risks; long-term safety and efficacy of erythropoietic stimulating agents
Paroxysmal nocturnal hemoglobinuria (PNH)	Complement inhibitors	Inhibition of the complement system to reduce red blood cell lysis	Approved treatments	Cost; risk of meningococcal infection

**Table 6:** New strategies for the treatment of acquired rare anemias.

Disease Target	Treatment Strategy	Mechanism/Approach	Development Stage	Key Considerations
Autoimmune hemolytic anemia (AIHA)	Novel immunomodulators	Targeting specific immune pathways (e.g., complement system, B cells) to reduce hemolysis	Clinical trials	Precision targeting to minimize side effects; long-term impact on immune system
Paroxysmal cold hemoglobinuria (PCH)	Complement inhibitors	Inhibition of the complement system to prevent red blood cell destruction	Clinical trials/approved for other indications	Limited treatment options; need for specific therapies
Atypical hemolytic uremic syndrome (aHUS)	Complement blockade	Targeting components of the complement system crucial in the pathogenesis of aHUS	Approved treatments	High cost; risk of infection; long-term treatment may be necessary
Pure red cell aplasia (PRCA)	Immunotherapy and thymectomy	Use of immunosuppressants; surgical removal of the thymus in cases associated with thymoma	Clinical practice/research	Risk of surgery; managing immunosuppression side effects
Drug-induced immune hemolytic anemia	Drug discontinuation and immunotherapy	Identification and discontinuation of causative drug; use of immunosuppressive drugs to control hemolysis	Clinical practice	Identifying the causative drug; balancing immune response while treating underlying condition
Anemia of chronic disease (ACD)	Iron reutilization agents	Agents that improve iron mobilization and utilization, targeting hepcidin or its regulators	Clinical trials	Addressing the underlying inflammatory condition; potential for broader application in chronic diseases
Infection-related hemolytic anemia	Antimicrobial therapy and supportive care	Direct treatment of the underlying infection; supportive care to manage anemia and prevent complications	Clinical practice	Effective infection control; managing hemolysis and anemia symptoms
